# Synergistic Efficacy of WST11-VTP and P-Selectin-Targeted Nanotherapy in a Preclinical Prostate Cancer Model

**DOI:** 10.3390/cancers17142361

**Published:** 2025-07-16

**Authors:** Lucas Nogueira, Ricardo Alvim, Hanan Baker, Karan Nagar, Jasmine Thomas, Laura Alvim, Kwanghee Kim, Daniel A. Heller, Augusto Reis, Avigdor Scherz, Jonathan Coleman

**Affiliations:** 1 Urology Service, Department of Surgery, Memorial Sloan Kettering Cancer Center, New York, NY 10065, USA; lucasnogueira@me.com (L.N.);; 2 School of Medicine, Department of Surgery, Federal University of Minas Gerais, Belo Horizonte 31270-901, Brazil; 3 Molecular Pharmacology Program, Memorial Sloan Kettering Cancer Center, New York, NY 10065, USA; 4 Department of Plants and Environmental Sciences, The Weizmann Institute of Science, Rehovot 7610001, Israel

**Keywords:** prostate cancer, tumor ablation, VTP, focal therapy, chemotherapy

## Abstract

**Objective:** Radical therapies are associated with significant morbidity in patients with localized prostate cancer (PCa). While advances in nuclear magnetic resonance techniques have enabled the development of focal ablation procedures that can selectively destroy tumors, preserve the gland and surrounding structures, and minimize side effects, existing vascular-targeted photodynamic therapy (VTP) and nanodrug therapies often face limitations, such as recurrence and insufficient drug concentration at the tumor site. This study investigated a novel approach that combines VTP with systemic treatment using drug-loaded nanoparticles in a murine model, demonstrating substantial advancements beyond current monotherapies. **Methods:** SCID (severe combined immunodeficiency) mice were engrafted with androgen-sensitive prostate tumor cells (LNCaP-AR) and treated with a combination of VTP and two different drugs linked to fucoidan nanoparticles (Enzalutamide and Paclitaxel). Experiments were performed using different cohorts: the evaluation of oncological effect, the administration time and concentration of systemic therapy, a comparison of efficacy between VTP and radiotherapy, and the induction of the abscopal effect in untreated synchronous tumors. **Results:** The groups that received combination therapy showed better tumor control. After eight weeks, the recurrence-free survival rates were 87.5%, 62.5%, and 50% in the VTP + N-PAC, VTP + N-ENZ, and VTP monotherapy groups, respectively (*p* < 0.05). There was a significant difference in the intra-tumoral concentration of nanodrugs between the groups with combined treatment and monotherapy. After two weeks, the monotherapy groups showed almost total elimination of the drugs, whereas in the combined therapy groups, this concentration remained high, starting to decrease after three weeks (*p* < 0.05). Treatment with nanodrugs associated with VTP showed superior oncological benefits compared to radiotherapy alone or in combination with other therapies. The abscopal effect on synchronous tumors was not demonstrated with VTP alone or in combination with nanodrugs. **Conclusions**: Combining vascular photodynamic therapy with nanodrugs was highly effective in treating a prostate tumor model, leading to increased survival and a reduced risk of tumor recurrence. This approach significantly advances beyond existing VTP and nanodrug therapies by improving tumor control, ensuring sustained intra-tumoral drug concentration, and yielding superior oncological outcomes. Our results suggest that this therapy is a potential treatment option for prostate tumors treated with VTP in future clinical trials.

## Simple Summary

This study introduces a new combination therapy to improve vascular-targeted photodynamic therapy (VTP) for prostate cancer. The main idea was that VTP-triggered inflammation increases P-selectin expression on tumor blood vessels, making it a target for drug-loaded nanoparticles. In a preclinical mouse model, WST11-VTP was combined with P-selectin-targeting nanoparticles carrying either paclitaxel or enzalutamide. The results showed a strong synergy, with the combination therapy significantly enhancing local tumor control and increasing recurrence-free survival compared to VTP or nanodrug monotherapies. In vivo imaging confirmed that VTP treatment resulted in a higher and more sustained concentration of nanoparticles within the tumor, validating the targeting mechanism. This approach, where VTP guides nanoparticle delivery, offers a promising strategy for future clinical trials in prostate cancer.

## 1. Introduction

Prostate cancer (PCa) is the most common solid tumor in men, with 1,466,680 cases and 396,792 deaths worldwide reported by 2022 [1]. The recent progress on screening policies and increased public awareness has changed PCa presentation. Approximately 70% of cases are diagnosed at the localized stage, and treatment offers high chances of successful oncological outcomes [2].

Treatment strategies for localized PCa are determined based on the assessment of risk, ranging from active surveillance for those at low risk of progression to more aggressive treatments, such as radical prostatectomy and radiation therapy, for high-risk cases. However, these approaches may have adverse effects and long-term implications. Active surveillance strategies, for instance, may lead to delayed treatment for initially classified low- or intermediate-risk localized diseases, which could result in disease progression and metastasis in a small percentage of patients. Conversely, radical prostatectomy and radiotherapy, when combined with androgen deprivation therapy (ADT), may lead to complications that can significantly impact a patient’s quality of life, such as impotence and incontinence [3,4].

Prostate cancer is one of the few types of solid tumors treated with whole-organ treatment. Owing to advancements in imaging techniques, aggressive tumors can now be identified more accurately, leading to the development of focal therapies to reduce treatment-related complications [5]. An example of such a minimally invasive treatment is vascular-targeted photodynamic (VTP) therapy, which eliminates cancer cells by targeting the vascular compartment of the tumor while preserving healthy tissue [6,7]. VTP is a minimally invasive tissue ablation modality that targets the tumor vasculature. In this modality, laser light delivered at a specific wavelength activates a photosensitizing substance (Padeliporfin, WTS-11, TOOKAD Soluble, Steba Biotech, France). Following intravenous administration, WST-11 binds non-covalently to albumin. It is sequestered within the circulatory system, with a half-life of less than 60 min in humans, thereby avoiding long-term skin toxicity. The excessive generation of reactive oxygen species upon light activation of WST11 confirms immediate hypoxia, which activates nitric oxide (NO) radical generation. The co-generation of nitric oxide and oxygen radicals generates peroxynitrite, leading to targeted tissue destruction through direct cytotoxicity, vascular shutdown, and the activation of an immune response [8,9].

WST11-VTP has demonstrated effectiveness in several animal and clinical genitourinary cancer [10,11,12,13] models. Currently, it is being evaluated in Phase III clinical trials to treat upper tract urothelial carcinoma (NCT04620239) [14]. VTP therapy using WST11 (WST11-VTP) has demonstrated significant clinical benefits as a localized gland ablation therapy for low-risk prostate cancer. Azzouzi et al. published a prospective randomized phase III study comparing WTS11-VTP to active surveillance in patients with low-risk prostate cancer. A total of 413 patients were randomly assigned to WTS11-VTP (n = 206) or active surveillance (n = 207). After a median follow-up of 2 years, patients undergoing WTS11-VTP had a lower rate of disease progression (28% vs. 58%, HR: 0.34), a higher rate of negative biopsies (49% vs. 14%, HR: 3.67), and a lower need for radical treatment. (6% vs. 29%, *p* < 0.0001). Analysis of the International Index of Erectile Function (IIEF-15) and International Prostate Symptom Score (IPSS) questionnaires showed a deterioration in erectile and voiding functions in the first 6 months; however, after 2 years of follow-up, there was no statistically significant difference in functional outcomes compared with the active surveillance group [15]. This study led to the approval of WTS11-TVF for the treatment of low-risk localized prostate cancer in Europe. Preliminary results from a prospective phase II study of WST11-VTP treatment in patients with favorable intermediate-risk prostate cancer revealed that 82% of patients exhibited an absence of Gleason Grade Group 2 disease after the first treatment. Twenty-two percent required retreatment with hemiablation. Among patients who completed a one-year follow-up after WST11-VTP, 83% showed no Gleason pattern 4 or 5 on biopsy [10]. However, other studies have reported disease recurrence in 27% of patients with low- and intermediate-risk PCa in control biopsies at 12 and 24 months after WST11-VTP [16].

WST11-VTP therapy is associated with an intense inflammatory reaction in the treatment environment, where the resulting inflammatory infiltrate plays an essential role in tumor ablation. In this environment, P-selectin, an inflammatory cell adhesion molecule responsible for leukocyte recruitment and platelet attachment [17], is continuously expressed in endothelial cells and stored in intracellular granules known as Weibel–Palade bodies [18]. After endothelial activation, as part of the inflammatory reaction, P-selectin translocates to the cell membrane and lumen of the blood vessels, allowing leukocyte passage to the interstitium [19].

Shamay et al. developed nanoparticles based on fucoidan, a polysaccharide derived from algae, with a high affinity for P-selectin. Researchers have reported that these nanoparticles can transport drugs such as taxanes, which are known to act on prostate tumor cells. Furthermore, they demonstrated that radiotherapy results in the local overexpression of P-selectin, leading to improved oncological outcomes when combined therapy is used. The same group demonstrated P-selectin expression in contralateral non-treated tumors [20]. 

This study introduces a novel approach by combining vascular-targeted photodynamic therapy (VTP) with nanoparticle-delivered chemotherapy and androgen deprivation therapy to enhance treatment efficacy for localized prostate cancer. We hypothesized that the inflammation induced by WST11-VTP increases P-selectin expression in the tumor microenvironment, thereby improving the targeted delivery and retention of P-selectin-binding nanodrugs. This research is significant as it addresses the limitations of current monotherapies, such as tumor recurrence and inadequate drug concentration, by proposing a synergistic strategy. We also compared this combination therapy against radiotherapy. We assessed its potential to induce systemic antitumor effects, aiming to establish a more effective treatment paradigm for future clinical applications in prostate cancer.

## 2. Methodology

### 2.1. Ethical Aspects

All animal procedures adhered to the ethical guidelines of the Memorial Sloan Kettering Cancer Center (MSKCC, New York, NY, USA), where they were carried out. The project and protocol for this study were approved by the Institutional Review Board and the Institutional Animal Care Committee (11-02-004).

### 2.2. Cell Culture

Lymph Node Carcinoma of the Prostate (LNCaP)-AR androgen receptor (AR) is a cell line that is responsive to androgenic action and has been utilized in all experiments. Another significant feature of this cell line was the absence of P-selectin overexpression. LNCaP-AR cells were cultured in RPMI medium supplemented with 10% fetal bovine serum (Life Technologies/Thermo Fisher Scientific, Waltham, MA, USA).

### 2.3. Preparation of Nanoparticles Loaded with Enzalutamide or Paclitaxel

Fucoidan, extracted from Fucus vesiculosus (20–300 kDa; Sigma), was diluted in diethyl-p-phenylenediamine and dissolved in bi-distilled water. The solution was homogenized at a 1:1 ratio, resulting in the formation of gel aggregates. These aggregates were collected after centrifugation (15,000× *g*, 10 min) and suspended in PBS (phosphate-buffered saline) containing excess fucoidan. The mixture was homogenized again using an ultrasonic probe tip (Sonics & Materials) at a 40% intensity for 10 s until a clear dark-red solution was formed. The material was then pelleted by centrifugation (30,000× *g*, 30 min), resuspended in PBS, and homogenized in a bath for 10 min.

In a microcentrifuge tube, the aqueous phase was prepared by combining 450 μL of fucoidan (10 mg mL^−1^), 100 μL of IR-783 (2 mg mL^–1^), and 100 μL of 0.01 mM sodium bicarbonate. The mixture was gently vortexed, and 50 μL of Enzalutamide or Paclitaxel (20 mg mL^–1^, dissolved in DMSO) was added dropwise. Following the complete addition of the organic phase, the resultant mixture was centrifuged for 15 min at 30,000× *g* to pellet the nanoparticles. Pellets were resuspended in 200 μL of deionized water or saline and further diluted as needed.

A spectrophotometer was used to quantify the encapsulation efficiency. The Paclitaxel and Enzalutamide nanoparticles measured 105 ± 4.2, 216.3 ± 8.6 nm in diameter, respectively, and exhibited a z-potential of approximately −55 mV (surface charge). Electron microscopy showed relatively uniform spherical morphologies. The nanoparticles exhibited good serum stability over 5 days and pH-dependent drug release rates, and they could be reconstituted after lyophilization.

### 2.4. In Vivo Studies

All the experiments utilized SCID (severe combined immunodeficiency) mice aged seven to eight weeks (Frederick National Laboratory for Cancer Research, Frederick, MD, USA). Male mice were selected because hormonal fluctuations have less of an effect on them than on females. SCID mice have a compromised immune system, which allows for better evaluation of oncological results without interference from the animal’s defense mechanisms.

All mice were grafted onto the right flank with 1 × 10^6^ androgen-sensitive tumor cells from the LNCaP-AR line. In cohort 2, in which contralateral non-treated tumors were also assessed, the same number of cells was grafted on the left flank, although no active treatment was performed in this area. Before tumor implantation, the mice were anesthetized with inhaled isoflurane, meloxicam (2 mg/kg), and buprenorphine (0.5 mg/kg), and the hair and skin over the right flank were sterilized with a solution of iodopovidone and ethyl alcohol.

The mice were evaluated weekly for their health status and tumor growth. The animals were randomly assigned to their respective study groups when their tumor volume reached approximately 70 mm^3^ (an average of 6 weeks). In cohort 1 (which assessed the oncological effects, administration timing, and concentration of systemic therapy in the tumor environment), the groups were organized as follows: control (n = 4), monotherapy with Enzalutamide nanoparticles (NENZ, n = 4), monotherapy with Paclitaxel nanoparticles (NPAC, n = 4), monotherapy with VTP (n = 8), a combination of NENZ and VTP (VENZ, n = 8), a combination of NPAC and VTP (VPAC, n = 8), and a combination of NENZ (administered 2 h before) and VTP (SD-NENZ, n = 8). The experiment was repeated to evaluate the oncological effects of photodynamic vascular therapy compared to radiotherapy and the induction of the contralateral response (cohort 2), where the same number of cells was grafted onto the left flank. In this cohort, the groups were organized as follows: control (n = 4), monotherapy with radiotherapy RT (n = 5), a combination of RT with Paclitaxel nanoparticles (RPAC, n = 5), a combination of RT with Enzalutamide nanoparticles (RENZ, n = 5), monotherapy with VTP (n = 7), a combination of VTP with Paclitaxel nanoparticles (VPAC, n = 8), and a combination of VTP with Enzalutamide nanoparticles (VENZ, n = 8).

#### 2.4.1. Vascular-Targeted Photodynamic Therapy (VTP)

**Figure 1:**
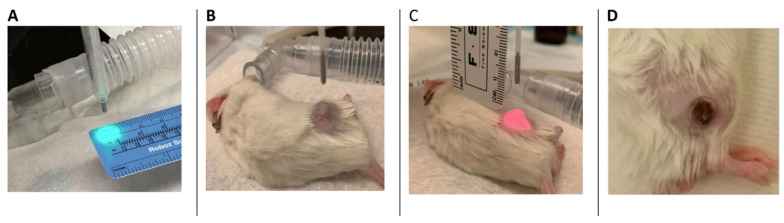
A preclinical prostate cancer model with VTP procedure. (**A**–**C**) A targeting system was used to ensure complete illumination of the target tumor with minimal exposure to the adjacent tissue. (**D**) An example of a healing scar over the previously seen tumor in a mouse that underwent complete response 14 days after VTP ablation. No residual tumor was present, and there was no damage to the surrounding tissue.

VTP was conducted six weeks after cell injection. The mice were anesthetized using inhaled isoflurane delivered through a well-fitted nasal cone. Following a retro-orbital injection of WST11 (9 mg/kg body weight), the tumors were illuminated with light at a wavelength of 753 nm, provided by a 600 μm fiber diode laser (Biolitec, East Longmeadow, MA, USA) for 10 min at 150 mW/cm^2^. VTP delivery and outcomes are depicted in Figure 1.

#### 2.4.2. Radiation Therapy (RT)

In patients subjected to radiotherapy, this procedure was administered six weeks after cell injection as a single dose of 5 Gy using an Xat 225 Cx microirradiator with a source-to-skin distance of 50 cm. The mice were sedated with intraperitoneal injections of ketamine (0.1 mg/g) and xylazine (0.02 mg/g). Only the tumor, surrounding skin, and subcutaneous tissues were exposed using a specialized lead template.

#### 2.4.3. Nanoparticles Administration

Fucoidan-based nanoparticles were synthesized at our institution using a previously described technique. The encapsulation of Enzalutamide and Paclitaxel included an infrared fluorophore (IR-783) to facilitate image generation via nanoprecipitation. Nanoparticle administration was performed 24 h before WST11-VTP through intraperitoneal injection at 5 mg/kg (Paclitaxel) and 50 mg/kg (Enzalutamide). In the group of mice (cohort 1) used to evaluate the effect of same-day administration, VTP was administered 2 h before VTP therapy.

#### 2.4.4. Imaging

Bioluminescent imaging using the IVIS Spectrum Optical Imaging System (Xenogen, Alameda, CA, USA) was performed weekly for four weeks following VTP administration in groups in which the concentration of systemic therapy in the tumor environment was assessed. Images were captured using a sensitive cooled camera (IVIS^®^, Xenogen). Image and signal quantification were managed by the acquisition and analysis software Living Image (Living Image^®^ Xenogen, version 4.5). The mice were anesthetized with 1–2.5% isoflurane and positioned inside a light-resistant chamber box, where they were continuously exposed to the anesthetic agent. The imaging times were varied from one second to two minutes. The emitted light levels were detected using an IVIS camera system, integrated, and displayed. Regions of interest in the displayed images were designated around the tumor sites to evaluate the signal intensity.

#### 2.4.5. Tumor Monitoring

The tumor volume was measured, and animal health was monitored at least once a week for up to 70 days after treatment. For lesion measurement, the tumors in cohort 1 were measured using a digital caliper, whereas a digital measurer (Peira TM900, Komax, Belgium) was used for cohort 2. When tumors exceeded 2000 mm^3^, the mice were euthanized with 100% carbon dioxide at 5 PSI for a minimum of 3 min. This euthanasia practice adhered to the Recommended Methods for Laboratory Animals, as outlined by the institution’s Research Animal Resources Center and the American Veterinary Medical Association Guidelines for the Euthanasia of Animals.

### 2.5. Statistical Analysis

Statistical analysis was performed using the GraphPad Prism^®^ program (GraphPad Software, version 10, San Diego, CA, USA). The study of the concentration of systemic therapy in the tumor environment was performed with the ANOVA test. The Kruskal–Wallis test was used to evaluate the differences in tumor evolution between all groups, and the t-test was used to compare the means between groups and each other. Survival rates were assessed using the Kaplan–Meier method, and differences in survival between groups were compared using the log-rank test. A value of *p* < 0.05 was defined as significant.

## 3. Results

### 3.1. The Evaluation of the Oncological Effect of the Association Between VTP and Nanoparticles Carrying Paclitaxel or Enzalutamide, Including the Administration Time

To evaluate the antitumor therapeutic effect of combining WST11-VTP treatment with nano-drugs, allograft-bearing mice were treated with WST11-VTP at week 6 after tumor implantation. As previously described, nanoparticles loaded with Paclitaxel and Enzalutamide were injected the day prior. Three of the 40 mice (7.5%) treated with WST11-VTP died within the first 72 h owing to the intense inflammatory reaction resulting from the treatment and were excluded from the study.

**Figure 2:**
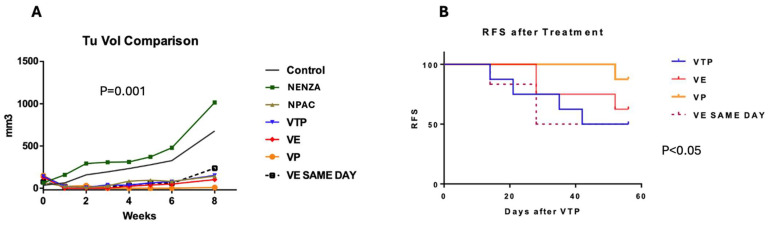
(**A**) Tumor volume progression after treatment (average, mm^3^). A comparison of the mean tumor volume in groups receiving nanoparticle (NENZA, NPAC) monotherapy and those undergoing combined therapy with nano-drugs (WST11-VTP: WST11-VTP, VE: WST11-VTP + NENZ, VP: WST11-VTP + NPAC) and the injection of nanoparticles loaded with Enzalutamide (VE SAME DAY) on the same day. WST11-VTP, associated with nanoparticles, was associated with improved tumor control. (**B**) The relapse-free survival curve for the groups receiving combined therapy with nano-drugs (VTP: WST11-VTP, VE: WST11-VTP + NENZ, VP: WST11-VTP + NPAC) and nanoparticles injected on the same day as Enzalutamide (VE SAME DAY). WST11-VTP, associated with nanoparticles, was associated with improved tumor control.

Compared to the controls, the combined therapy groups (WST11-VTP associated with nanoparticles) showed significantly delayed tumor growth (*p* < 0.0001), but no difference was observed between the different drugs (*p* = 0.9). Additionally, the combination therapy had lower recurrence rates than the monotherapy (nanoparticles or WST11-VTP; *p* < 0.05). Tumor growth occurred in only one mouse (12.5%) treated with WST11-VTP and Paclitaxel nanoparticles (Figure 2 and Figure S1). The injection of the nanodrug one day or two hours before treatment demonstrated the same efficacy in the NENZ groups. After eight weeks, the recurrence-free survival rates were 87.5%, 62.5%, and 50% in the WST11-VTP + NPAC, WST11-VTP + NENZ, and WST11-VTP monotherapy groups, respectively (*p* < 0.05) (Figure 2).

### 3.2. The Evaluation of the Intra-Tumor Concentration Time of Nanoparticles Carrying Paclitaxel or Enzalutamide

**Figure 3:**
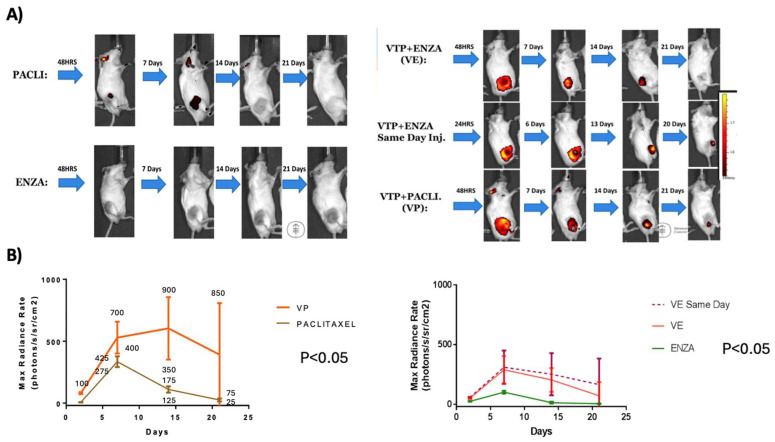
Comparison of images between nano-drug groups with and without associated VTP treatment: (**A**) IVIS images over time after administering nano-drugs with and without associated WST11-VTP. (**B**) Quantitative analysis of IVIS images comparing evolution of these groups (VE: WST11-VTP +NENZ).

The analysis of the intra-tumoral concentration of nano-drugs utilized the same cohort as mentioned in Section 1. A comparison of the bio-luminescent images revealed a significant difference in nano-drug concentrations within the tumor between the combined treatment and nanoparticle monotherapy groups. Overall, mice that received WST11-VTP along with nanodrugs exhibited higher and more sustained drug concentrations in the tumor environment compared to those that received nanodrugs alone. In the Paclitaxel groups, the average Max Radiance Rate (photons/s/sr/cm^2^) on days 7, 14, and 21 was 400 vs. 500, 100 vs. 550, and 20 vs. 450 for the nanodrug monotherapy and the nanodrugs combined with WST11-VTP groups, respectively (*p* < 0.05). In the Enzalutamide groups, the average radiance measurements on days 7, 14, and 21 were 90 vs. 250, 10 vs. 180, and 10 vs. 10 photons/s/sr/cm^2^ for the nanodrug monotherapy and the nanodrugs combined with the WST11-VTP groups, respectively (*p* < 0.05) (Figure 3 and Figure S2).

### 3.3. An Evaluation and Comparison of the Oncological Effects Between VTP and Radiation Therapy

To evaluate the antitumor therapeutic effect of the combination of WST11-VTP treatment and nanodrug radiotherapy, allograft-bearing mice were treated with VTP or RT six weeks after tumor implantation. As previously described, nanoparticles carrying Paclitaxel and Enzalutamide were injected on the previous day. Four of the 38 mice (10.5%) treated with WST11-VTP or radiation died in the first 72 h owing to the intense inflammatory reaction caused by the treatment and were excluded from the study.

**Figure 4:**
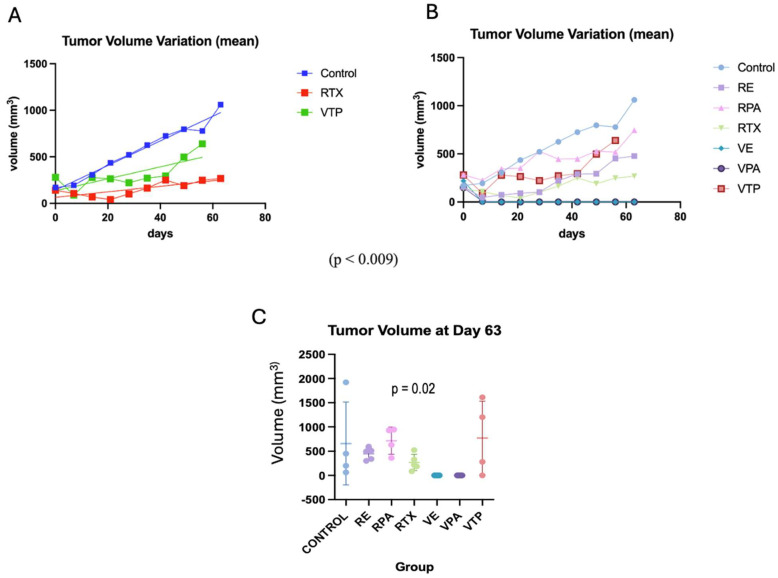
The progression of the tumor volume after treatment (average) compared with radiotherapy. (**A**) A comparison of the mean tumor volume between the VTP monotherapy and radiotherapy groups (VTP: WST11-VTP). (**B**) A comparison of the mean tumor volume in the nano-drug combination therapy groups (VTP: WST11-VTP; RE: RTX + ENZ; VE: WST11-VTP + NENZ; VPA: WST11-VTP + NPAC). (**C**) The tumor volume for each group (mean, SD) at day 63.

Compared to the controls, the monotherapy groups (WST11-VTP, RT) showed significantly delayed tumor growth (*p* < 0.009). However, despite a trend favoring the RT group, no significant difference was observed between the different regimens (*p* = 0.08). Regarding the associated therapy groups, when evaluated separately from the controls, there was a significant benefit for those who received WST11-VTP combined with Paclitaxel and Enzalutamide (*p* < 0.001). The paired analysis between the associated therapy groups demonstrated a benefit from the use of WST11-VTP compared to radiation therapy (VPAC vs. RPAC, *p* < 0.0001; VPAC vs. RENZ, *p* = 0.02; VENZ vs. RPAC, *p* < 0.0001; VENZ vs. RENZ, *p* = 0.01). No significant differences were found when analyzing the RENZ vs. RPAC or VENZ vs. VPAC groups (*p* = 0.8) (Figure 4 and Figure S3).

**Figure 5:**
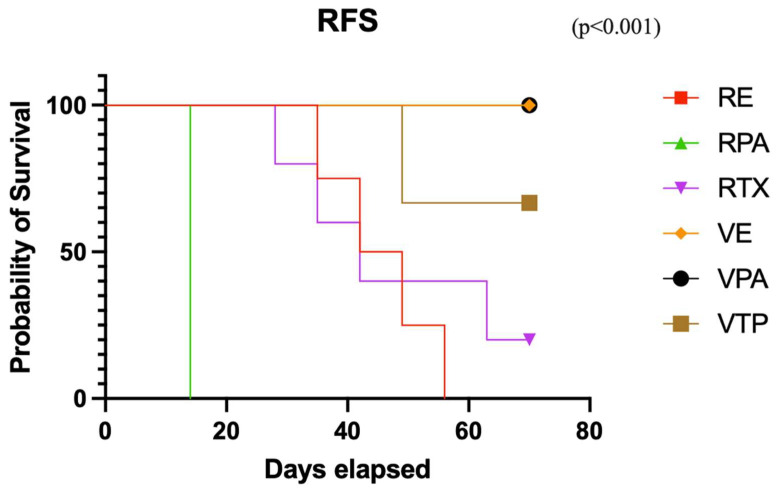
The relapse-free survival curve in the combined therapy groups with nanodrugs and radiotherapy (VTP: WST11-VTP, RE: RTX + ENZ, VE: WST11-VTP + NENZ, VPA: WST11-VTP + NPAC, RPA: RTX + NPAC).

The initial treatment response rates were 100%, 75%, 80%, 67%, 100%, and 100% in the RT, RPAC, RENZ, VTP, VPAC, and VENZ groups, respectively. The complete response rates in the RT, RPAC, RENZ, VTP, VPAC, and VENZ groups were 60%, 0%, 60%, 25%, 100%, and 100%, respectively. Notably, no evidence of disease (NED) was observed in any mice in the VENZ and VPAC groups at the end of the experiment, whereas recurrence occurred in all mice in the remaining treatment groups. The recurrence-free survival rate at the end of the experiment, calculated for mice that showed an initial response to therapy, was 20%, 0%, 0%, 67%, 100%, and 100% in the RT, RPAC, RENZ, VTP, VPAC, and VENZ groups, respectively (*p* < 0.001) (Figure 5).

### 3.4. Evaluation of Induction and Oncological Efficacy of VTP and Radiotherapy in Synchronous Untreated Contralateral Tumors

**Figure 6:**
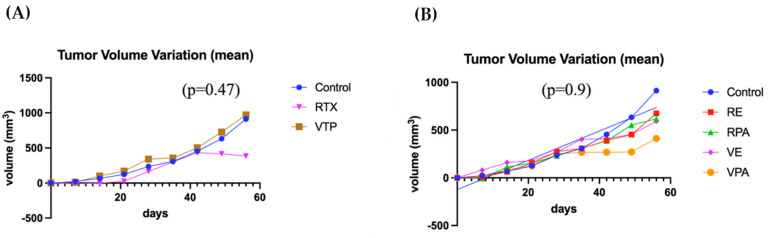
The progression of tumor volume variation in untreated contralateral tumors. (**A**) A comparison of the mean tumor volume variation between the groups receiving V WST11-VTP monotherapy and contralateral radiotherapy (VTP: WST11-VTP). (**B**) A comparison of the mean tumor volume variation in the combined therapy groups with nanodrugs in contralateral tumors (RE: RTX + ENZ, VE: WST11-VTP + NENZ, VPA: WST11-VTP + NPAC, RPA: RTX + NPAC).

**Figure 7:**
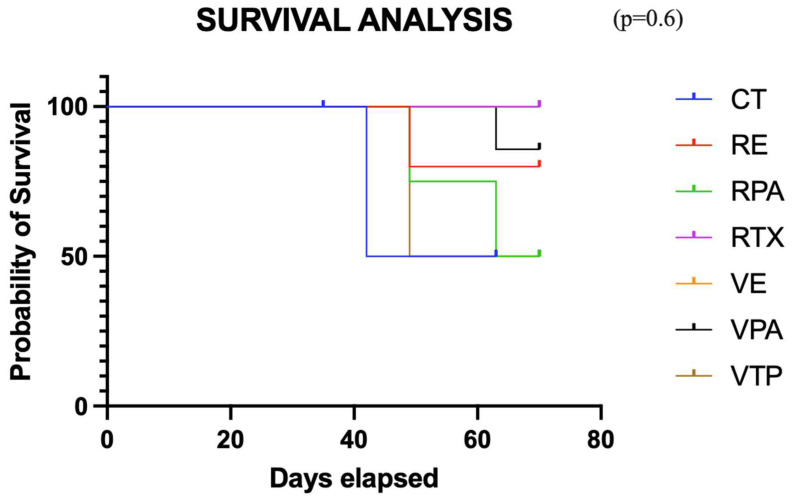
The survival curve for the combined therapy groups using nanodrugs and radiotherapy (VTP: WST11-VTP, RE: RTX + ENZ, VE: WST11-VTP + NENZ, VPA: WST11-VTP + NPAC, RPA: RTX + NPAC).

This analysis utilized the same cohort as in item 3. Compared to the controls, the monotherapy groups (VTP and RT) did not exhibit a decrease in tumor volume variation (*p* = 0.47), despite the trend observed in favor of the RT group from the seventh week onward. When evaluated separately from the controls, no significant benefit was demonstrated in the associated therapy groups (*p* = 0.9) (Figure 6). However, the mean tumor size in the group that received RT monotherapy remained stable after day 40, suggesting an oncological effect. Seven mice were sacrificed (control = 1, RENZ = 1, RPAC = 2, VPAC = 1, VTP = 2) because of the tumor volume limit (2000 mm^3^). The survival rates of the control, RT, RPAC, RENZ, VTP, VPAC, and VENZ groups at the end of the experiment were 67%, 100%, 67%, 80%, 71.5%, 87.5%, and 100%, respectively (Figure 7).

## 4. Discussion

Our study highlights the use of nanoparticles for drug delivery as a promising approach to enhance the efficacy of VTP treatment. We showed that WST11-VTP ablation combined with P-selectin-targeted nanoparticle therapy, along with local Enzalutamide and Paclitaxel, reduced tumor burden and improved survival more effectively than WST11-VTP alone in a mouse model of prostate cancer. Furthermore, we demonstrated that oncological outcomes are superior to those achieved by combining these nanoparticle-delivered agents with radiation therapy.

Loaded nanoparticles are an emerging strategy for cancer treatment, primarily focusing on enhancing the delivery and efficacy of anticancer therapies. Fucoidan-based nanoparticles have garnered significant attention due to their enhanced biological activity and the ability to target P-selectin, a cell adhesion molecule that is overexpressed in various tumors [21]. These nanoparticles were designed to improve the selective delivery of therapeutic agents to cancer cells, thereby increasing the drug concentration at the tumor site. Nanoparticles modified with sulfates, such as BRAF-mutated melanomas and BRCA-mutated breast cancers, have been developed to actively target P-selectin-expressing cancers. These nanoparticles have shown enhanced accumulation in tumor tissues, improving therapeutic efficacy and safety in both in vitro and in vivo models [22]. Shamay et al. evaluated P-selectin nanoparticles as targets for localized drug delivery to tumor sites. They synthesized a nanoparticle carrier for chemotherapeutic drugs and targeted therapeutics using the algae-derived polysaccharide fucoidan, which has affinity for P-selectin. These fucoidan-based nanoparticles target the activated endothelium, demonstrating their ability to penetrate endothelial barriers and show a therapeutic advantage over untargeted chemotherapeutics in vivo [20]. Furthermore, they indicated that radiation increased endothelial P-selectin expression and particle accumulation in a tumor model that did not express P-selectin, resulting in antitumor efficacy. Jafari et al. developed fucoidan–doxorubicin nanoparticles to target P-selectin, enhancing the delivery and cytotoxicity of doxorubicin specifically to cancer cells with high P-selectin expression, such as the MDA-MB-231 breast cancer cell line, thus minimizing the side effects associated with doxorubicin [23].

Our results demonstrate that the enhanced efficacy and improved drug retention of the combined therapy stem from a targeted, mechanism-based interaction between VTP and the nanoparticle formulations. The therapeutic action of WST11-VTP is known to induce an intense, localized inflammatory response [17]. This inflammation, driven by cytokines such as histamine and thrombin, triggers the upregulation and translocation of P-selectin—a cell adhesion molecule—to the surface of endothelial cells lining the tumor vasculature. Our nanoparticles were formulated with fucoidan, a polysaccharide with a high affinity for P-selectin, allowing them to actively bind to this VTP-induced molecular target.

This targeted adhesion, likely coupled with VTP-induced vascular permeability, facilitates the migration of the nanoparticles into the tumor’s interstitial space. Our IVIS imaging results confirmed this enhanced migration and permanence; one week after administration, the average nanodrug concentration in the combined therapy groups was double that of the monotherapy groups. Moreover, bioluminescence demonstrated that the nanoparticles remained present for up to three weeks, thereby enhancing their efficacy. This sustained presence shows that the nanoparticles are not merely passively accumulating but are actively captured and retained at the site of action, creating a local drug depot that enhances treatment effectiveness. The synergy achieved is analogous to that of Antibody-Drug Conjugates (ADCs), a class of targeted therapies that combine the specificity of monoclonal antibodies with the potent effects of chemotherapy drugs, and have shown significant results in various cancers, including breast, lung, and urothelial tumors [24,25].

The association between WST-11VTP and systemic ADT has already been evaluated in preclinical studies that demonstrated that VTP can potentiate the effects of ADT in prostate cancer xenografts. This combination leads to the significant inhibition of tumor growth compared with either therapy alone, suggesting a synergistic effect that could be beneficial in treating high-risk prostate cancer tumors [26]. The efficacy of this combination was demonstrated in both phases of the study. WST11-VTP, in association with nanoparticles, decreased tumor growth and led to lower recurrence rates. Notably, recurrence did not occur in the mice receiving WST11-VTP or nanoparticles in the second cohort. Our study also extends the range of therapies for which ADT and chemotherapy have been shown to synergize. WST11-VTP, in conjunction with these treatments, showed similar results to their association with radiation therapy, indicating a potential role for this strategy in patients with more aggressive disease, which requires multimodal treatment for better outcomes.

The tumor model utilizing a cell line that does not express P-selectin demonstrated that radiation increased endothelial P-selectin expression and nanoparticle accumulation, leading to antitumor efficacy. The same study revealed P-selectin expression in non-irradiated tumors in irradiated mice, resembling the abscopal effect, a radiotherapy-induced distant immune activation resulting in tumor regression at non-irradiated sites [20]. Our study found no significant benefit in the associated therapy groups when evaluated separately from the control groups. However, the mean tumor size of the group that received RT monotherapy remained stable after day 40. Similar results were not observed in the VTP monotherapy or combination groups. One reason for this lack of efficacy is that SCID mice have a compromised immune system and cannot induce an abscopal response [27].

## 5. Conclusions

Combining WST11-VTP and P-selectin-targeted nanoparticles presents a synergistic approach for precision medicine. P-selectin-targeted nanoparticles can deliver drugs directly to the tumor vasculature, enhancing the selectivity and efficacy of VTP. Additionally, vascular damage caused by WST11-VTP can further upregulate P-selectin expression, creating a positive feedback loop that boosts nanoparticle accumulation. This dual strategy may improve cancer therapeutic outcomes. Our results suggest that this therapy may be a viable treatment option for prostate tumors undergoing VTP in future clinical trials.

## Data Availability

The data presented in this study are available on request from the corresponding author due to the institution’s data release protocol.

## Supplementary Materials

The following supporting information can be downloaded at: https://www.mdpi.com/article/10.3390/cancers17142361/s1, Figure S1: Individual tumor volume progression after treatment (average, mm^3^) by group; Figure S2: Quantitative analysis of IVIS images comparing the luminescence (Max Radiance Rate (photons/s/sr/cm^2^)) evolution of each group; Figure S3: Mean tumor volume (average, mm^3^) progression after treatment by group.
