# Supplementary material for: Synergistic Efficacy of WST11-VTP and P-Selectin-Targeted Nanotherapy in a Preclinical Prostate Cancer Model
Source: Cancers (Basel). 2025 Jul 16;17(14):2361. doi: 10.3390/cancers17142361 (PMC12293855; doi:10.3390/cancers17142361)
Supplement: Supplementary file 1 [file cancers-17-02361-s001.zip › cancers-3710106-supplementary.pdf]

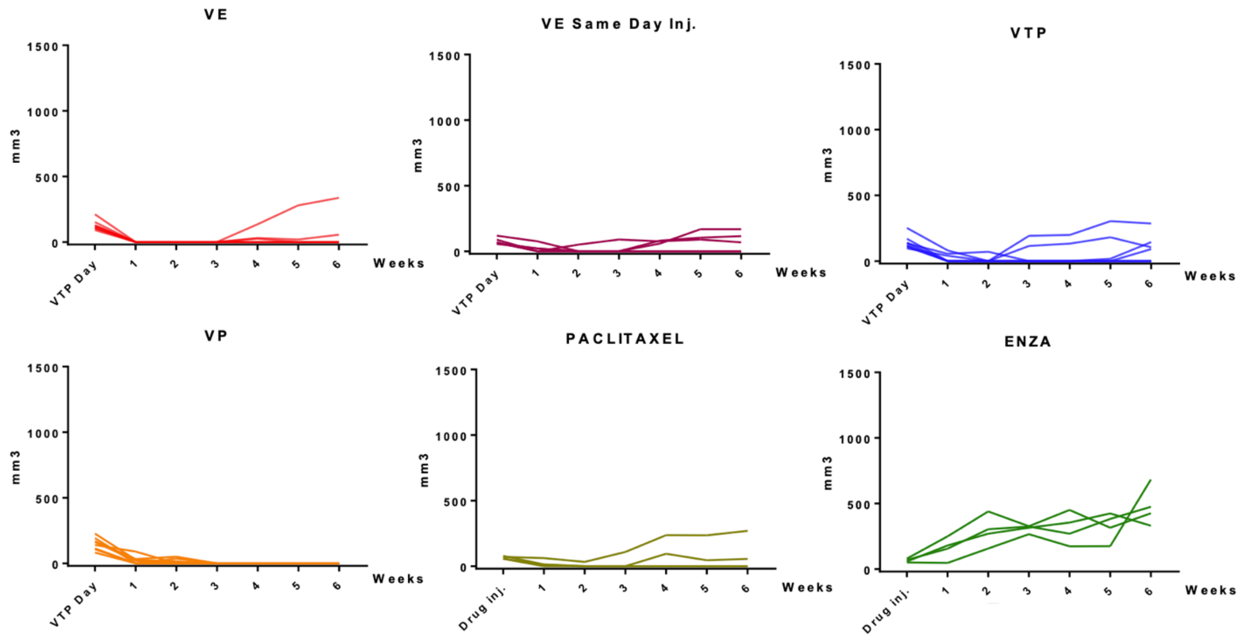

Figure S1. Individual tumor volume progression after treatment (average, mm^3) by group.

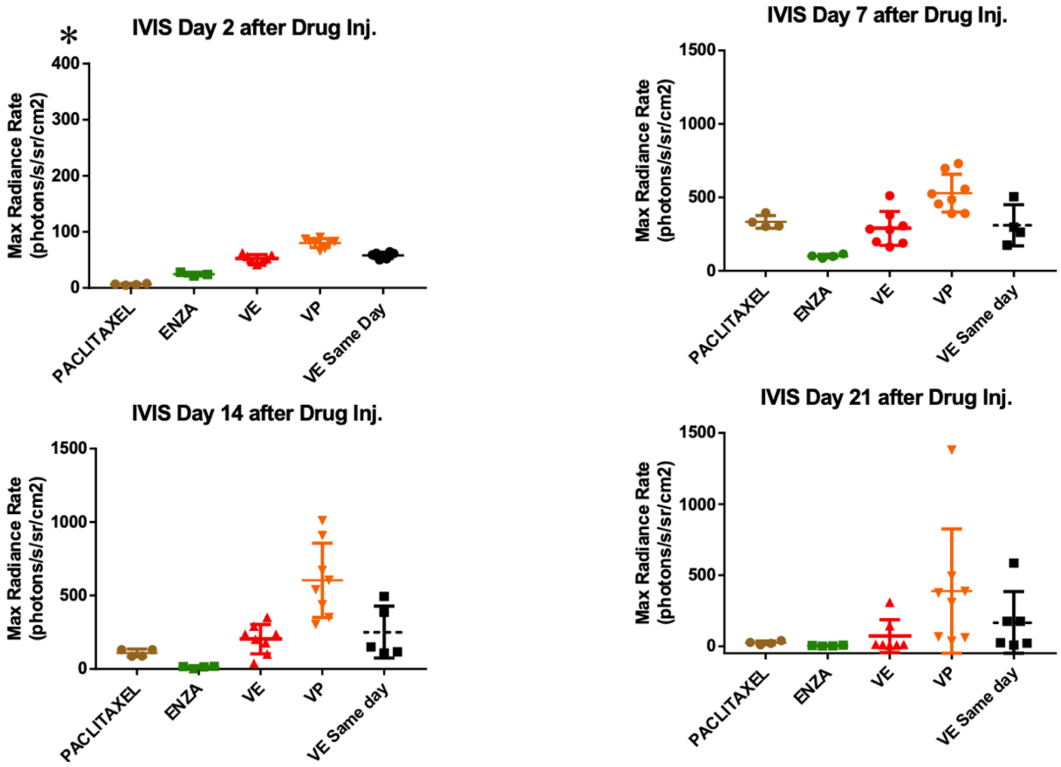

Figure S2. Quantitative analysis of IVIS images comparing the luminescence (Max Radiance Rate (photons/s/sr/cm2)) evolution of each group.

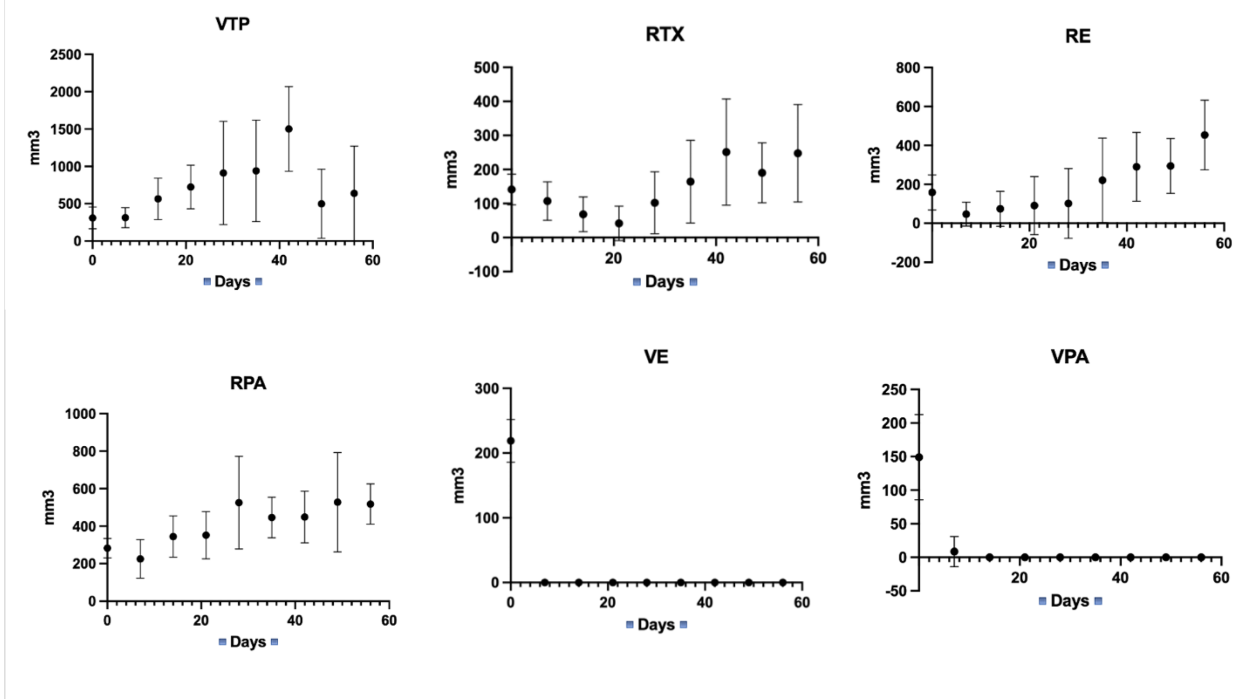

Figure S3. Mean tumor volume (average, mm^3) progression after treatment by group.
